# A possible contribution of the locus coeruleus to arousal enhancement with mild exercise: evidence from pupillometry and neuromelanin imaging

**DOI:** 10.1093/texcom/tgad010

**Published:** 2023-06-08

**Authors:** Yudai Yamazaki, Kazuya Suwabe, Atsuko Nagano-Saito, Kousaku Saotome, Ryuta Kuwamizu, Taichi Hiraga, Ferenc Torma, Kenji Suzuki, Yoshiyuki Sankai, Michael A Yassa, Hideaki Soya

**Affiliations:** Laboratory of Exercise Biochemistry and Neuroendocrinology, Faculty of Health and Sport Sciences, University of Tsukuba, 1-1-1 Tannoudai, Tsukuba, Ibaraki 305-8574, Japan; Sport Neuroscience Division, Advanced Research Initiative for Human High Performance (ARIHHP), Faculty of Health and Sport Sciences, University of Tsukuba, 1-1-1 Tennoudai, Tsukuba, Ibaraki 305-8574, Japan; Sport Neuroscience Division, Advanced Research Initiative for Human High Performance (ARIHHP), Faculty of Health and Sport Sciences, University of Tsukuba, 1-1-1 Tennoudai, Tsukuba, Ibaraki 305-8574, Japan; Faculty of Health and Sport Sciences, Ryutsu Keizai University, 120 Ryugasaki, Ibaraki 301-0844, Japan; Center for Cybernics Research, University of Tsukuba, 1-1-1 Tennoudai, Tsukuba, Ibaraki 305-8573, Japan; Laboratory of Exercise Biochemistry and Neuroendocrinology, Faculty of Health and Sport Sciences, University of Tsukuba, 1-1-1 Tannoudai, Tsukuba, Ibaraki 305-8574, Japan; Sport Neuroscience Division, Advanced Research Initiative for Human High Performance (ARIHHP), Faculty of Health and Sport Sciences, University of Tsukuba, 1-1-1 Tennoudai, Tsukuba, Ibaraki 305-8574, Japan; Department of Radiology, Ushiku Aiwa General Hospital, 896 Inoko-cho, Ushiku, Ibaraki 300-1296, Japan; Center for Cybernics Research, University of Tsukuba, 1-1-1 Tennoudai, Tsukuba, Ibaraki 305-8573, Japan; Department of Radiological Sciences, School of Health Sciences, Fukushima Medical University, 1 Hikarigaoka, Fukushima, Fukushima 960-1295, Japan; Laboratory of Exercise Biochemistry and Neuroendocrinology, Faculty of Health and Sport Sciences, University of Tsukuba, 1-1-1 Tannoudai, Tsukuba, Ibaraki 305-8574, Japan; Graduate School of Letters, Kyoto University, Sakyo-ku, Kyoto 606-8501, Japan; Laboratory of Exercise Biochemistry and Neuroendocrinology, Faculty of Health and Sport Sciences, University of Tsukuba, 1-1-1 Tannoudai, Tsukuba, Ibaraki 305-8574, Japan; Laboratory of Exercise Biochemistry and Neuroendocrinology, Faculty of Health and Sport Sciences, University of Tsukuba, 1-1-1 Tannoudai, Tsukuba, Ibaraki 305-8574, Japan; Sport Neuroscience Division, Advanced Research Initiative for Human High Performance (ARIHHP), Faculty of Health and Sport Sciences, University of Tsukuba, 1-1-1 Tennoudai, Tsukuba, Ibaraki 305-8574, Japan; Center for Cybernics Research, University of Tsukuba, 1-1-1 Tennoudai, Tsukuba, Ibaraki 305-8573, Japan; Center for Cybernics Research, University of Tsukuba, 1-1-1 Tennoudai, Tsukuba, Ibaraki 305-8573, Japan; Sport Neuroscience Division, Advanced Research Initiative for Human High Performance (ARIHHP), Faculty of Health and Sport Sciences, University of Tsukuba, 1-1-1 Tennoudai, Tsukuba, Ibaraki 305-8574, Japan; Department of Neurobiology and Behavior, University of California, Irvine, CA 92679-3800, United States; Center for the Neurobiology of Learning and Memory, University of California, Irvine, CA 92679-3800, United States; Laboratory of Exercise Biochemistry and Neuroendocrinology, Faculty of Health and Sport Sciences, University of Tsukuba, 1-1-1 Tannoudai, Tsukuba, Ibaraki 305-8574, Japan; Sport Neuroscience Division, Advanced Research Initiative for Human High Performance (ARIHHP), Faculty of Health and Sport Sciences, University of Tsukuba, 1-1-1 Tennoudai, Tsukuba, Ibaraki 305-8574, Japan

**Keywords:** arousal, locus coeruleus, neuromelanin, pupil diameter, very light-intensity exercise

## Abstract

Acute mild exercise has been observed to facilitate executive function and memory. A possible underlying mechanism of this is the upregulation of the ascending arousal system, including the catecholaminergic system originating from the locus coeruleus (LC). Prior work indicates that pupil diameter, as an indirect marker of the ascending arousal system, including the LC, increases even with very light-intensity exercise. However, it remains unclear whether the LC directly contributes to exercise-induced pupil-linked arousal. Here, we examined the involvement of the LC in the change in pupil dilation induced by very light-intensity exercise using pupillometry and neuromelanin imaging to assess the LC integrity. A sample of 21 young males performed 10 min of very light-intensity exercise, and we measured changes in the pupil diameters and psychological arousal levels induced by the exercise. Neuromelanin-weighted magnetic resonance imaging scans were also obtained. We observed that pupil diameter and psychological arousal levels increased during very light-intensity exercise, which is consistent with previous findings. Notably, the LC contrast, a marker of LC integrity, predicted the magnitude of pupil dilation and psychological arousal enhancement with exercise. These relationships suggest that the LC-catecholaminergic system is a potential a mechanism for pupil-linked arousal induced by very light-intensity exercise.

## Introduction

Physical activity, represented by aerobic exercise, has beneficial effects on the brain and mental health (Cotman et al. 2007; Hillman et al. 2008). Many studies have focused on the effect of exercise performed at a moderate or higher intensity and have shown beneficial effects. On the other hand, our research group has been elucidating the effect of mild exercise, below the intensity that initiates stress-related responses (Soya et al. 2007), on brain function in a series of translational studies beginning with animals and shifting to humans. In humans, even very light-intensity exercise (defined as <37% $\dot{\text{V}}$O_2peak_ by the American College of Sports Medicine: ACSM; ACSM 2022) can stimulate the brain and improve prefrontal cognition and hippocampal memory (Byun et al. 2014; Suwabe et al. 2018) through activation in associated brain regions. Very light-intensity exercise is useful because it can be performed without eliciting stress responses (Soya et al. 2007; Hill et al. 2008) or causing a decrease in adherence (Perri et al. 2002; Ekkekakis et al. 2008). Therefore, understanding the mechanism of the beneficial effects of very light-intensity exercise is required.

The prefrontal cortex and hippocampus receive the projection of multiple neuromodulators, such as monoamine and acetylcholine, mainly from the ascending arousal system (Ungerstedt 1971; Bland and Oddie 1998; Zaborszky et al. 2015). It has been speculated that the activation of the ascending arousal system during exercise is a factor in improving cognitive function (McMorris and Hale 2014; McMorris 2016). These ideas lead to the hypothesis that an increase in arousal level may play a role in cognitive enhancement with very light-intensity exercise. Indeed, we have demonstrated that even very light-intensity exercise robustly enhances psychological arousal, and this enhancement is associated with an improvement in cognitive function (Byun et al. 2014; Suwabe et al. 2018). However, psychological arousal is only a subjectively expressed index, and there is no direct evidence that the ascending arousal system is activated during very light-intensity exercise. Furthermore, it remains unknown whether the upregulation of psychological arousal, which is associated with the improvement of cognitive function, is mediated by the ascending arousal system in humans. Thus it is fruitful if there is supporting evidence connecting the psychological and physiological parameters.

The use of pupillometry may shed light on these questions. Pupil diameter, under constant brightness, is believed to reflect the arousal state (Bradshaw 1967). Importantly, recent animal studies have rapidly accumulated evidence that pupil fluctuation allows the precise tracking of the firing of the locus coeruleus (LC) (Joshi and Gold 2020; Privitera et al. 2020), a key region of the ascending arousal system (Aston-Jones et al. 1991; Breton-Provencher and Sur 2019). The LC has two modes of activity, phasic and tonic, and both activities are thought to be reflected by the pupil diameter (Joshi et al. 2016; Hayat et al. 2020). Additionally, recent human studies have also shown that pupil fluctuation is associated with LC activity (Murphy et al. 2014), suggesting the usefulness of pupil diameter in tracking LC activity in humans. Our previous study showed that pupil diameter increases even during very light-intensity exercise and that this is correlated with exercise-induced enhancement of psychological arousal (Kuwamizu et al. 2022). This evidence strengthens our hypothesis that exercise, even very light-intensity, stimulates the ascending arousal system, in particular the LC, the center of this system, and pupil diameter could be an indirect marker that allows us to capture this activation even during exercise.

Unfortunately, it remains unclear whether exercise-induced pupil dilation is related to LC activity. Although animal studies have confirmed that exercise activates the LC neurons (Yanagita et al. 2007; Rodovalho et al. 2020), it is challenging to directly measure the LC activity during exercise in humans due to its size and anatomical position (Fernandes et al. 2012) and to body movement during exercise. To address this issue, adopting neuromelanin sensitive-magnetic resonance imaging (MRI) may be helpful. When using specialized MRI sequences, T1-weighted images of the LC show a hyperintense signal (LC contrast) (Enochs et al. 1997; Trujillo et al. 2017). LC contrast is associated with the distribution of the catecholaminergic neurons in the LC (Keren et al. 2015) and the concentration of neuromelanin (Cassidy et al. 2019), suggesting that it reflects the neuronal integrity and density of catecholaminergic neurons in the LC (Sasaki et al. 2006; Keren et al. 2009, 2015). Indeed, LC contrast is reduced in Alzheimer’s disease (Takahashi et al. 2015) and major depression (Shibata et al. 2008; Sasaki et al. 2010), where LC neurodegeneration occurs, and correlates with cognitive function in older adults (Clewett et al. 2016; Dahl et al. 2019). Not only that, LC contrast also is linked to the LC activity measured by the BOLD signal during memory encoding with arousal-evoking stimuli (Clewett et al. 2018) and with the pupil response to an odd-ball task (Mather et al. 2020). Given these findings, LC contrast reflects the structural integrity, but it would also be related to the LC activity. Exploring whether such a link can be observed between LC contrast and exercise-induced pupil response may provide an important suggestion for the contribution of the LC to arousal enhancement evoked by very light-intensity exercise. It has been confirmed that LC contrast has high reproducibility even on different measurement dates (Wengler et al. 2020). Therefore, it was thought that this study could be conducted with reliability ensured even if the day of the measurement of LC contrast and exercise-induced arousal response were different.

In this study, we aimed to clarify whether the change in arousal state assessed by pupil dilation and evoked by very light-intensity exercise is associated with LC contrast measured in a different day. We hypothesized that exercise-induced pupil dilation and enhancement of psychological arousal would be correlated with LC contrast.

## Materials and Methods

### Participants

We recruited 22 young males in this study. This study was approved by the Institutional Ethics Committee of the University of Tsukuba and was in accordance with the Declaration of Helsinki. Written informed consent was obtained from all participants. None of the participants had any history of neurological, psychiatric, or cardiovascular illnesses or of visual abnormality, and all were nonsmokers. For the main experiment, 1 participant was unable to undergo an MRI scan due to a magnetic implant, resulting in data from 21 participants being used for the analyses (Table 1). On the first visit, participants answered questionnaires about their usual physical activity levels and depressive mood using the Japanese version of the International Physical Activities Questionnaire (IPAQ) and the Beck Depression Inventory-2 (BDI-2), respectively. Post hoc sensitivity analysis (*n* = 21, α = 0.05, Power = 0.8) showed that the sensitivity for detecting correlation effects was sufficient if it exceeded 0.542, as computed using G^*^Power version 3.1.9.6.

**Table 1:** Participant demographics.

	Values (mean ± SD)
Sample size	21
Age (years)	21.3 ± 2.0
Height (cm)	173.2 ± 5.6
Weight (kg)	65.0 ± 10.6
BMI (kg/m^2^)	21.6 ± 3.1
$\dot{\text{V}}$ O_2peak_ (mL/kg/min)	43.8 ± 7.8
HR_peak_ (bpm)	178.7 ± 11.4
WR_peak_ (Watt)	232.9 ± 37.3
IPAQ_TPA (METs-min/week)	4280.3 ± 4375.3
BDI-2	7.6 ± 3.9

BMI, body mass index; $\dot{\text{V}}$O_2peak_, peak oxygen uptake; HR_peak_, peak heart rate; WR_peak_, peak work rate.

### Procedure

In this study, participants visited the laboratory for a total of 4 times. The first visit was for a graded maximal exercise test. The second and third visits were for the acute exercise or resting control to be performed outside of the MRI scanner to measure the change in the pupil diameter and psychological arousal. The order in which participants underwent each condition was randomized. Structural images and LC contrast were scanned at the fourth visit. All visits were separated by at least 48 h. The average interval of measurement days between exercise-induced arousal and LC contrast was 9.7 ± 6.8 days. Participants were instructed to refrain from exhaustive exercise and alcohol consumption for 24 h before the experiment, from caffeine on the experimental days and from eating for 2 h before the experiment.

### Graded maximal exercise test

The graded maximal exercise test was conducted to measure each individual’s peak oxygen uptake ($\dot{\text{V}}$O_2peak_) and to determine the exercise workload for each participant. Participants performed incremental exercise to exhaustion using a recumbent ergometer based on our previous studies. After 3 min of warm-up at 30 W, the workload was increased linearly by 20 W per minute until the participant could no longer maintain the required pedaling rate. The pedaling rate was maintained at 60 rpm using a digital metronome. Exhaustion was determined when the participant’s pedaling rate dropped below 55 rpm for longer than 5 s. Exhaled gas, including $\dot{\text{V}}$O_2_, $\dot{\text{V}}$CO_2_, and VE, were continuously measured using a gas analyzer (Aeromonitor AE280S, Minato Medical Science, Japan) at a sampling rate of 0.2 Hz. The respiratory exchange ratio (RER) was calculated as the $\dot{\text{V}}$CO_2_/$\dot{\text{V}}$O_2_ ratio. Heart rate (HR) was measured using a Polar monitor (RS800CX, Polar Electro Oy, Finland). The rate of perceived exertion (RPE) was measured every 1 min using Borg’s scale, ranging from 6 to 20 (Borg 1982). $\dot{\text{V}}$O_2peak_ was considered to have been reached when 2 of the following criteria were satisfied: (i) RER reached above 1.10; (ii) 90% of age-predicted peak HR (220 - age) was achieved; and (iii) RPE exceeded 18. The exercise workload needed to achieve 30% $\dot{\text{V}}$O_2peak_ was estimated for each participant using the linear regression equations derived from the time series data of $\dot{\text{V}}$O_2_ and exercise workload.

### Acute aerobic exercise

**Fig. 1:**
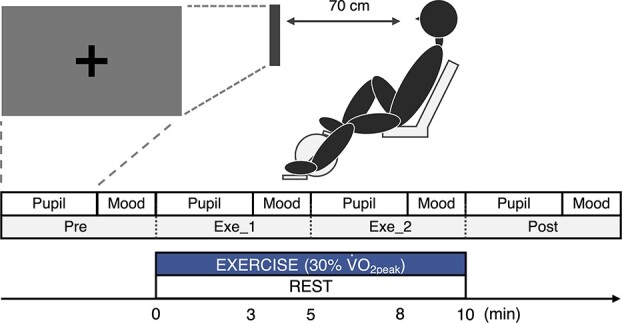
Experimental protocol for acute exercise.

In order to measure the arousal changes induced by very light-intensity exercise, as done in our previous studies, participants conducted an acute exercise and resting control outside of the MRI scanner in the second and third visits (Fig. 1). The brightness in the laboratory was in the range of 1,450–1,550 lx, as the same with our previous study (Kuwamizu et al. 2022). In the exercise condition, participants performed 10 min of pedaling exercise at an exercise load equivalent to 30% $\dot{\text{V}}$O_2peak_, which had been determined by the graded maximal exercise test, using a recumbent ergometer. The pedaling rate was maintained at 60 rpm using a digital metronome. Pupil diameter and psychological arousal were measured before, during, and after exercise. During the exercise, pupil diameter was measured at 3 min after which psychological arousal was assessed using a questionnaire (details of the methods are described below). In the control condition, participants performed measurements and questionnaires while sitting at rest on the ergometer.

### Measurement of pupil diameter

Pupil diameters during rest and exercise were recorded using a screen-based eye-tracker (Tobii pro nano, Tobii AB, Danderyd, Sweden) following the same method as described in our previous study (Kuwamizu et al. 2022). During measurement, a fixation cross appeared on the screen which was positioned 70 cm in front of participants. The fixation cross and the background color were black (RGB: 0, 0, 0) and gray (RGB: 120, 120, 120), respectively. Participants were instructed to gaze at the fixation cross without staring. Each measurement lasted for 3 min at a sampling rate of 60 Hz. Missing or invalid pupil data due to blinking or looking away from the screen were automatically removed by the analysis software (Tobii Pro Lab, Tobii AB, Danderyd, Sweden). Since our focus was on long-lasting pupil dilation during exercise, pupil diameter was averaged across the 3 min for both eyes at each time point, and these values were used for statistical analysis.

### Measurement of psychological arousal

Psychological arousal was measured using the Two-Dimensional Mood Scale (Sakairi et al. 2013). This questionnaire is used to assess an individual’s arousal and pleasure levels by measuring 8 items (energetic, lively, lethargic, listless, relaxed, calm, irritated, and nervous). Participants were asked to answer their current mood using a 6-point Likert scale ranging from 0 = “Not at all” to 5 = “Extremely.” Using these values, calculated arousal levels could range from −20 to 20.

### MRI acquisition

Structural images (MPRAGE T1-weighted image, neuromelanin sensitive-weighted image) were taken at fourth visit. Imaging data were acquired on an Achieva 3.0 Tesla MRI scanner (Achieva-TX, Philips Healthcare, The Netherlands) with a 32-channel sensitivity encoding head coil at the center for Cybernics Research at the University of Tsukuba. We collected high-resolution structural images using an MPRAGE T1-weighted sequence with an FOV of 240 × 240 mm, repetition time of 12 ms, echo time of 5.9 ms, and flip angle of 9°, comprising 250 oblique slices with 0.65-mm isotropic resolution. After that, neuromelanin sensitive-weighted MRI scans were conducted using a T1-weighted turbo spin echo imaging with an FOV of 200 × 178 × 29 mm (AP, RL, FH), repetition time of 503 ms, echo time of 13 ms, total scan duration of 9 min and 56 s, flip angle of 90°, 10 average to increase SNR, 9 axial slices, 174.6 Hz of bandwidth, voxel size of 0.5 × 0.62 mm^2^, slice thickness of 3 mm, slice gap of 3.3 mm, and reconstruction matrix of 1,024.

### Analysis of LC contrast

**Fig. 2:**
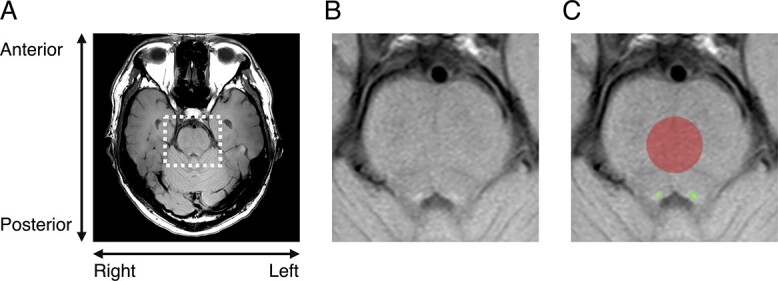
Individual examples of LC position. A) The whole image of a single participant measured by neuromelanin sensitive-weighted MRI. B) Enlarged view of brainstem area from (A). C) VOI of LC (green) and PT (red) superimposed on (B).

Using a neuromelanin-sensitive T1-weighted structural scan, both sides of the LC were semiautomatically segmented using MRIcron software (https://www.nitrc.org/projects/mricron) and these were identified as voxels of interest (VOIs) (Fig. 2). Additionally, a radiologist (AN-S) visually confirmed that the LC was included in the VOI for all participants. We averaged the mean signal intensities (SIs) from both sides of the VOI and used this average value to calculate the LC contrast. A reference VOI was located in the pontine tegmentum (PT) with a radius of 7 mm. The LC contrast was calculated using a formula: (SI_LC_ − SI_PT_)/SD_PT_ (Chen et al. 2014; Isaias et al. 2016), where SD_PT_ represents the standard deviation of the signal in the PT. The median value of LC contrast was 2.33 (range: 1.22–7.54).

### Statistical analysis

Statistical analysis was performed using SPSS statistics ver. 27 (IBM Corp., Armonk, NY, United States). First, we confirmed that all data were normally distributed using the Shapiro–Wilk test. The LC contrast was log-transformed because it was not normally distributed. Pupil diameter and psychological arousal measured by the second and third visits were analyzed using 2-way repeated measures analysis of variance (rmANOVA) with the factors of condition (exercise and control) and time (pre, Exe_1, Exe_2, and post). If the assumption of sphericity was violated in Mauchly’s sphericity test, the degree of freedom was corrected using the Greenhouse–Geisser correction, and *F* and *P* values were then recalculated. When the main effects or interactions were identified, Bonferroni’s corrected multiple comparison was conducted as the post hoc test.

We investigated the relationships between exercise-induced change in pupil dilation, psychological arousal, and LC contrast. Exercise-induced change in pupil diameter and psychological arousal for both conditions were calculated by subtracting the value for preexercise from the averaged during-exercise value. Then, the difference in values between the exercise and control conditions was used for analysis. Pearson’s correlation analysis was conducted for calculated pupil diameter, psychological arousal, and LC contrast. As shown in Table 1, the physical aspects and depressive mood states of participants in this study were slightly varied (range of $\dot{\text{V}}$O_2peak_: 31.9–62.5; range of total physical activity [TPA]: 66–18,360; range of BDI-2: 2–19). In order to confirm whether these variabilities affect the relationships among parameters, we also conducted a partial correlation analysis that controlled $\dot{\text{V}}$O_2peak_, TPA, and BDI-2 as covariates. The statistical significance level was set at *P* = 0.05. All data are represented as mean ± standard error of mean.

## Results

### Confirmation of exercise intensity

We checked whether the pedaling exercise was performed within the range of our physical targets. The average HR and RPE at the end of exercise were 101.0 ± 2.2 bpm and 9.8 ± 0.3 points, respectively. These were within the range of very light-intensity exercise according to the ACSM guidelines (Liguori et al. 2022) and were approximately the same level as in our previous studies (Byun et al. 2014; Suwabe et al. 2018).

### Change in pupil diameter and psychological arousal induced by very light-intensity exercise

**Fig. 3:**
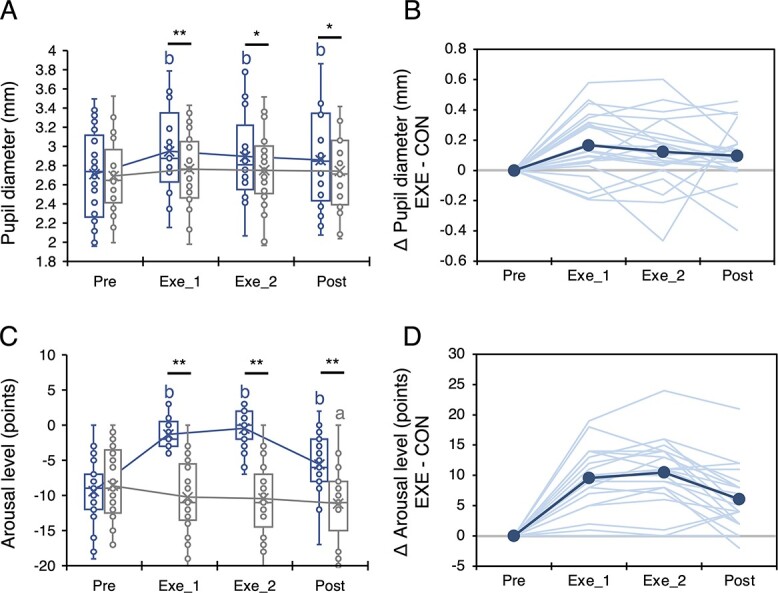
Changes in pupil diameter and psychological arousal. A) and C) A time series of pupil dilation and psychological arousal, respectively. B) and D) Individual changes in pupil diameter and psychological arousal ([Exe − Pre in exercise condition] − [Exe − Pre in control condition]). Note that (B) and (D) are only included to visually show changes in parameters; statistical analysis was conducted using raw data. Blue and gray lines represent the exercise and control conditions, respectively. ^*^: *P* < 0.05 and ^*^^*^: *P* < 0.01 between conditions. a: *P* < 0.05 and b: *P* < 0.01 compared to Pre.

Figure 3 shows the time series data for pupil diameter and psychological arousal. The rmANOVA revealed an interaction of condition and time for both pupil diameter (*F*_(3, 60)_ = 4.675, *P* = 0.005, η^2^*P* = 0.189) and psychological arousal (*F*_(2.159, 43.17)_ = 44.843, *P* < 0.001, η^2^*P* = 0.692). Post hoc multiple comparison with Bonferroni’s correction revealed that very light-intensity exercise significantly increased the pupil diameter and psychological arousal (all were *P* < 0.05), as in our previous studies (Byun et al. 2014; Suwabe et al. 2018; Kuwamizu et al. 2022).

### Relationships between exercise-induced pupil dilation, psychological arousal, and LC contrast

**Fig. 4:**
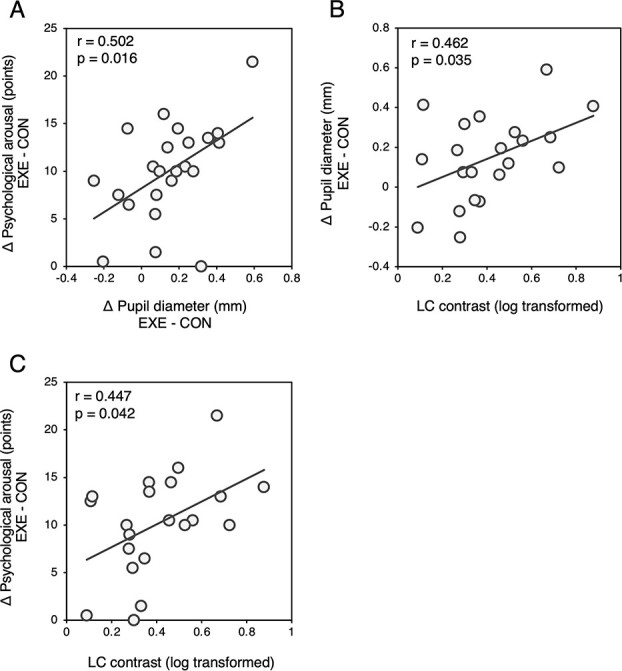
Correlations between A) change in psychological arousal and change in pupil diameter, B) change in pupil diameter and LC contrast, and C) change in psychological arousal and LC contrast. Exercise-induced pupil diameter and psychological arousal is calculated as the difference in values of the averages for Exe_1 and Exe_2 and Pre.

Figure 4 shows the correlation among exercise-induced pupil dilation, psychological arousal, and LC contrast. As in our previous study (Kuwamizu et al. 2022), exercise-induced pupil dilation was correlated with the exercise-induced increase in psychological arousal (*r* = 0.502, *P* = 0.016). In addition, both values were correlated with LC contrast (pupil: *r* = 0.462, *P* = 0.035; arousal: *r* = 0.447, *P* = 0.042). When participants’ physical ($\dot{\text{V}}$O_2peak_, TPA) and psychological (BDI-2) aspects were controlled as covariates, these relationships became more pronounced (pupil dilation − psychological arousal: pr = 0.620, *P* = 0.006; pupil dilation − LC contrast: pr = 0.660, *P* = 0.003; psychological arousal − LC contrast: pr = 0.498, *P* = 0.035). However, the baseline pupil diameter and psychological arousal (average values of Pre in both conditions) did not correlate with LC contrast (pupil: *r* = −0.119, *P* = 0.641; psychological arousal: *r* = 0.102, *P* = 0.662) even when the physical and psychological aspects were controlled (pupil: pr = −0.104, *P* = 0.682; psychological arousal: pr = 0.063, *P* = 0.805).

## Discussion

### Main findings

In this study, we aimed to explore the relationships among exercise-induced pupil dilation, psychological arousal, and LC integrity. As previously demonstrated, even very light-intensity exercise reliably increased the pupil diameter and psychological arousal. Additionally, we found that exercise-induced pupil dilation and psychological arousal were positively correlated. Consistent with our hypothesis, the change in these values induced by exercise positively correlated with the LC contrast. These results demonstrate, for the first time, that LC integrity is associated with pupil dilation and enhancement of psychological arousal evoked by 10 min of very light-intensity exercise.

### Exercise-induced pupil dilation and psychological arousal

First, we confirmed that the exercise intensity was in the “very-light” range according to ACSM guidelines (Liguori et al. 2022) based on the HR and RPE at the end of the exercise sessions. We also confirmed that acute, very light-intensity exercise led to increased pupil dilation and enhanced psychological arousal, as previously reported in our studies (Byun et al. 2014; Suwabe et al. 2018; Kuwamizu et al. 2022). Furthermore, pupil dilation during exercise was correlated with the enhancement of psychological arousal, which is consistent with our previous study using incremental exercise (Kuwamizu et al. 2022). Our previous study (Kuwamizu et al. 2022) and the present study suggest that an enhancement of psychological arousal induced by very light-intensity exercise, without stress-related responses, may be due to the activity of ascending arousal system, which is predicted by pupil dynamics.

### Relationships between LC contrast and exercise-induced arousal

The LC is a core component of the ascending arousal system and plays a key role in attention and arousal by projecting neurons throughout the entire cortex (Aston-Jones and Cohen 2005). Since several animal studies have shown that LC is activated by wheel running (Yanagita et al. 2007) and moderate-intensity treadmill running to exhaustion (Rodovalho et al. 2020), it has been speculated that LC is stimulated by exercise and contributes to the enhancement of arousal levels induced by exercise. Additionally, our previous studies using humans have indicated that very light-intensity exercise enhances psychological arousal (Byun et al. 2014; Suwabe et al. 2018) and increase pupil diameter (Kuwamizu et al. 2022), the indirect marker of the LC. While these results led to the hypothesis that exercise, even very light-intensity exercise, stimulates the LC, there was a lack of firm evidence for that. In this study, we demonstrated for the first time that the structural integrity of the LC is correlated with the pupil dilation and enhancement of psychological arousal evoked by very light-intensity exercise even measured on a different day. As mentioned above, pupil diameter precisely tracks the LC activity (Liu et al. 2017; Hayat et al. 2020) and is used as an indirect marker of the ascending arousal system (Joshi and Gold 2020; Privitera et al. 2020). On the other hand, psychological arousal is subjectively expressed and is possibly caused by the modulation of cortical states via projections from the ascending arousal system, including the LC. Although LC contrast is a structural index, and these measurements were conducted on a different day, our results suggest that the structural integrity of the LC might link to the responsiveness of the LC itself or of the ascending arousal system induced by acute exercise. LC contrast decreases in patients with major depression and Alzheimer’s disease (Shibata et al. 2008; Takahashi et al. 2015) who have LC dysfunction (Arango et al. 1996; Germain et al. 2007; Braak et al. 2011). These patients have also been shown to exhibit abnormal pupil responses (Silk et al. 2007; Granholm et al. 2017; Jiménez et al. 2021). Recent studies have also shown that the LC contrast is positively correlated with LC activation during encoding with a threat (Clewett et al. 2018) and with the pupil dilation evoked by an odd-ball task (Mather et al. 2020). Furthermore, MRI contrast in the substantial nigra is associated with the responsiveness of dopamine release induced by amphetamine administration (Cassidy et al. 2019). These findings support that structural integrity is partially related to its function and responsiveness. However, the result should be interpreted carefully, as it has several potential limitations, which are described below. In contrast to the exercise-induced response, pupil diameter and psychological arousal at baseline were not correlated with the LC contrast. This suggests that the structural integrity of the LC may be related to its response rather than the basal state. However, the possibility that the pupillometry and questionnaires used in this study are better suited for detecting changes in the state, rather than the basal state, should be considered.

There were relatively large individual differences in the physical activity level and depressive mood state among the participants in this study. However, even when these factors were controlled for as covariates, the relationships between pupil dilation, enhancement of psychological arousal, and LC contrast remained. Given that, it is unlikely that the correlation obtained in this study was caused by individual differences in the physical activity and depressive mood state among the participants.

One thing to note is that several studies have indicated that LC contrast does not directly measure neuromelanin but rather reflects the magnetization transfer effect (Watanabe et al. 2019). It is still unclear whether LC contrast is directly related to neuromelanin accumulation and catecholaminergic activity that would precede it. Additionally, as this study focused on a structural perspective, we did not measure the actual activity of the LC during exercise. Future studies are needed to explore the direct relationship between responsiveness of the LC during exercise and changes in pupil dilation and psychological arousal. Very light-intensity exercise has been shown to enhance the arousal levels and coincidently improve prefrontal cognition and hippocampal memory function (Byun et al. 2014; Suwabe et al. 2018). It is also important to examine the role of LC integrity in the improvement of brain function following acute, very light-intensity exercise. We did not measure LC contrast and exercise-induced changes in arousal on the same day. Although this point was a potential limitation, the results obtained in this study can be interpreted as sufficiently valid and reliable due to the high reproducibility of LC contrast (Cassidy et al. 2019; Wengler et al. 2020).

## Conclusion

In this study, we, for the first time, demonstrated that pupil dilation and enhancement of psychological arousal induced by 10 min of very light-intensity exercise are correlated with the structural integrity of the LC. Although we could not directly examine the LC activity, the results in this study may provide important insights into the mechanisms of the benefits of very light-intensity exercise for improving cognitive and memory function.

## Supplementary Material

Supplementary_information_CCC-2023-00004_Final_ver_tgad010

## Data Availability

The data of this study are available from the corresponding author on reasonable request.
