# Supplementary material for: A possible contribution of the locus coeruleus to arousal enhancement with mild exercise: evidence from pupillometry and neuromelanin imaging
Source: Cereb Cortex Commun. 2023 Jun 8;4(2):tgad010. doi: 10.1093/texcom/tgad010 (PMC10267300; doi:10.1093/texcom/tgad010)
Supplement: Supplementary_information_CCC-2023-00004_Final_ver_tgad010 [file supplementary_information_ccc-2023-00004_final_ver_tgad010.docx]

**Supplementary information**

A Possible contribution of the locus coeruleus to arousal enhancement with mild exercise: Evidence from pupillometry and neuromelanin imaging

Yudai Yamazaki^1,2^, Kazuya Suwabe^2,3,4^, Atsuko Nagano-Saito^2,5^, Kousaku Saotome^4,6^, Ryuta Kuwamizu^1,7^, Taichi Hiraga^1^, Ferenc Torma^1,2^, Kenji Suzuki^4^, Yoshiyuki Sankai^4^, Michael A. Yassa^2,8,9^, Hideaki Soya^1,2^*

^1^Laboratory of Exercise Biochemistry and Neuroendocrinology, Faculty of Health and Sport Sciences, University of Tsukuba, Ibaraki, Japan

^2^Sport Neuroscience Division, Advanced Research Initiative for Human High Performance (ARIHHP), Faculty of Health and Sport Sciences, University of Tsukuba, Ibaraki, Japan

^3^Faculty of Health and Sport Sciences, Ryutsu Keizai University, Ibaraki, Japan

^4^Center for Cybernics Research, University of Tsukuba, 1-1-1 Tennoudai, Tsukuba, Ibaraki 305-8577, Japan

^5^Department of Radiology, Ushiku Aiwa General Hospital, Ibaraki, Japan

^6^Department of Medical Radiology Technology, School of Health Sciences, Fukushima Medical University, Fukushima, Japan

^7^Graduate School of Letters, Kyoto University, Japan

^8^Department of Neurobiology and Behavior, University of California, Irvine, USA

^9^Center for the Neurobiology of Learning and Memory, University of California, USA

*Address for correspondence: Hideaki Soya, Ph.D.

Laboratory of Exercise Biochemistry and Neuroendocrinology; Sport Neuroscience Division,

Advanced Research Initiative for Human High Performance (ARIHHP), Faculty of Health and Sport Sciences, University of Tsukuba, 1-1-1 Tennoudai, Tsukuba 305-8574, Ibaraki, Japan

E-mail: soya.hideaki.gt@u.tsukuba.ac.jp

Tel/Fax: +81-29-853-2620

**Confirmation of the validity of two-dimensional mood state to capture an intensity-dependent arousal enhancement during exercise**

Throughout our related studies, we have been using psychological arousal measured by Two-Dimensional Mood Scale (TDMS)(Sakairi et al. 2013) to determine the change in arousal level induced by acute exercise (Byun et al. 2014; Suwabe et al. 2018, 2021; Damrongthai et al. 2021; Kuwamizu et al. 2022). We already investigated an intensity-dependent arousal enhancement can be captured by TDMS during graded exercise (Kuwamizu et al. 2022), but not constant load exercise. To verify whether the questionnaire-based psychological arousal is effective for capturing the intensity-dependent elevation in an individual's arousal level even on the constant load exercise, we performed an additional experiment using a separate sample (twelve males, 21.5 ± 0.9 years). The additional experiment consisted of three conditions: high-intensity exercise (HIGH), very light-intensity exercise (VL), and resting control (CON). In the HIGH and VL conditions, participants performed 10 min of exercise using a recumbent ergometer with the intensity, which was determined with the graded maximal exercise test, set at 70% VO_2peak_ or 30% VO_2peak_, respectively. In the CON condition, participants sat on the ergometer instead of exercising. Psychological arousal was measured before, during (3 and 8 min after the start of the exercise), and after the exercise using TDMS. The detail explanation of TDMS is described in main text.

Two-way repeated measures analysis of variance (rmANOVA) with the factors of condition (HIGH, VL, and CON) and time (pre, Exe_1, Exe_2, post) for psychological arousal. The RPE at the end of exercise and the average HR during exercise were analyzed using one-way rmANOVA with the factor of condition.

The average HR during exercise for each condition was 167.33 ± 3.31 (HIGH), 106.67 ± 2.35 (VL), and 65.03 ± 1.89 (CON) (F_(2,22)_ = 288.066, p < 0.001). The RPE at the end of exercise for each condition was 17.17 ± 0.52 (HIGH), 9.17 ± 0.42 (VL), and 6.25 ± 0.18 (CON) (F_(2,22)_ = 671.416, p < 0.001). Psychological arousal levels increased with exercise in an intensity-dependent manner (condition: F_(2,22)_ = 28.04, p < 0.001; time: F_(2.133,23.465)_ = 17.815, p < 0.001; condition × time: F_(6,66)_ = 13.838, p < 0.001; Table. S1). Based on these results, we determined that the measured psychological arousal achieved in this and previous studies adequately reflects the change in an individual arousal state induced by exercise.

**Table S1. Changes in psychological arousal by exercise with different intensities**

|  | Pre | Exe_1 | Exe_2 | Post |
| --- | --- | --- | --- | --- |
| HIGH | -5.75±1.12^a^ | 2.42±0.57*^ab^ | 6.17±1.05*^ab^ | -0.67±1.47^a^ |
| VL | -6.33±1.13 | -2.58±0.83*^a^ | -0.67±0.92*^a^ | -5.83±1.27 |
| CON | -9.92±1.43 | -10.08±1.79 | -10.33±1.74 | -8.67±1.72 |

*: p < 0.05 vs Pre; a: p < 0.05 vs CON; b: p < 0.05 vs VL. HIGH: high-intensity exercise condition (70% VO_2peak_), VL: very light-intensity exercise condition (30% VO_2peak_), CON: resting control condition.

**References**

Byun K, Hyodo K, Suwabe K, Ochi G, Sakairi Y, Kato M, Dan I, Soya H. 2014. Positive effect of acute mild exercise on executive function via arousal-related prefrontal activations: An fNIRS study. Neuroimage. 98:336–345.

Damrongthai C, Kuwamizu R, Suwabe K, Ochi G, Yamazaki Y, Fukuie T, Adachi K, Yassa MA, Churdchomjan W, Soya H. 2021. Benefit of human moderate running boosting mood and executive function coinciding with bilateral prefrontal activation. Sci Rep. 11:1–12.

Kuwamizu R, Yamazaki Y, Aoike N, Ochi G, Suwabe K, Soya H. 2022. Pupil-linked arousal with very light exercise: pattern of pupil dilation during graded exercise. J Physiol Sci. 72:1–9.

Sakairi Y, Nakatsuka K, Shimizu T. 2013. Development of the Two-Dimensional Mood Scale for self-monitoring and self-regulation of momentary mood states: Mood scale for self-monitoring. Jpn Psychol Res. 55:338–349.

Suwabe K, Byun K, Hyodo K, Reagh ZM, Roberts JM, Matsushita A, Saotome K, Ochi G, Fukuie T, Suzuki K, Sankai Y, Yassa MA, Soya H. 2018. Rapid stimulation of human dentate gyrus function with acute mild exercise. Proceedings of the National Academy of Sciences. 115:10487–10492.

Suwabe K, Hyodo K, Fukuie T, Ochi G, Inagaki K, Sakairi Y, Soya H. 2021. Positive Mood while Exercising Influences Beneficial Effects of Exercise with Music on Prefrontal Executive Function: A Functional NIRS Study. Neuroscience. 454:61–71.
